# The impact of high-salt diet and diuretics on the development of the aestival phenomenon in patients with chronic heart failure

**DOI:** 10.3389/fnut.2025.1538962

**Published:** 2025-07-22

**Authors:** Dmitrii O. Dragunov, Anna V. Sokolova, Vadim M. Mitrokhin, Grigory P. Arutyunov

**Affiliations:** ^1^Federal State Autonomous Educational Institution of Higher Education “N.I. Pirogov Russian National Research Medical University” of the Ministry of Health of the Russian Federation, Moscow, Russia; ^2^Research Institute for Healthcare Organization and Medical Management of Moscow Healthcare Department, Moscow, Russia

**Keywords:** chronic heart failure, diuretics, sodium intake, aestivation, osmolytes, urea, plasma osmolality

## Abstract

**Background:**

Chronic heart failure (CHF) often requires the use of high-dose loop diuretics to achieve decongestion. However, such therapy may lead to adverse effects, including deterioration of renal function and electrolyte imbalance. Recent evidence suggests that diuretic-induced dehydration may trigger metabolic responses resembling aestivation—a survival mechanism involving a shift from inorganic to organic osmolytes, particularly increased urea synthesis, to conserve water.

**Methods:**

This prospective, single-center cohort study included 102 patients (median age 75 years, 57.8% female) hospitalized with CHF from January to July 2023. The diuretic group received average daily doses of furosemide 39.1 ± 22.1 mg, torasemide 7.4 ± 3 mg, and spironolactone 42 ± 12.4 mg. Biochemical parameters—including sodium, potassium, glucose, urea, and estimated plasma osmolality (eOSM)—were assessed on days 1 and 7 of hospitalization. Plasma osmolyte ratios (PropUrea/eOSM, PropNa/eOSM) were calculated. Propensity score matching (PSM) was used to adjust for confounders such as age, ejection fraction, and renal function.

**Results:**

By day 7, plasma osmolality in the diuretic group increased from 300 [297; 304] to 302.2 [298.3; 305.8] mOsm/L (*p* = 0.039), while no significant change occurred in the non-diuretic group. Urea levels rose to 7.95 [5.65; 9.90] mmol/L in the diuretic group versus 5.90 [5.05; 7.50] mmol/L in the control group (*p* = 0.012). The PropUrea/eOSM increased to 2.63% [1.89; 3.28] in the diuretic group compared to 2.00% [1.70; 2.50] (p = 0.011). Conversely, PropNa/eOSM decreased to 46.46% [46.02; 46.74] versus 46.68% [46.33; 46.89] (*p* = 0.050). Multivariate logistic regression confirmed that diuretic therapy was independently associated with these changes: PropUrea/eOSM (OR = 3.52, 95% CI: 1.94–7.26, *p* < 0.001), and PropNa/eOSM (OR = 0.16, 95% CI: 0.06–0.39, *p* < 0.001). These effects were most pronounced in patients consuming >10 g/day of salt.

**Conclusion:**

This study demonstrated that in patients with chronic heart failure (CHF), intensive loop diuretic therapy—especially when combined with high sodium intake—is associated with a shift in plasma osmolytes, marked by increased urea and reduced sodium contributions to osmolality. These changes suggest activation of water-conservation mechanisms and are independent of CHF severity or renal dysfunction, as confirmed by propensity score matching. Clinically, the urea-to-osmolality ratio may serve as an early marker of metabolic stress and muscle catabolism. Patients consuming >10 g/day of salt appear especially susceptible to this aestivation-like response. Early identification of these changes may guide adjustments in diuretic regimens, as well as prompt nutritional and physical interventions to mitigate sarcopenia and functional decline. These findings support a personalized approach to diuretic therapy in CHF, emphasizing the role of dietary sodium in shaping metabolic responses and highlighting metabolic aestivation as a potential contributor to fatigue and weakness in this population.

## 1 Introduction

Decompensated heart failure often requires the use of high doses of diuretics. However, research indicates that the administration of large doses of diuretics can lead to several side effects, including worsening renal function and electrolyte disturbances. The ADHERE study showed that patients receiving lower doses of furosemide (<160 mg) had a reduced risk of hospital mortality, shorter stays in the ICU, and fewer prolonged hospitalizations or renal side effects compared to patients receiving more than 160 mg of furosemide (1). The use of higher doses of diuretics was associated with increased diuresis and more favorable outcomes at some secondary endpoints, but also posed a higher risk of deterioration of renal function (2). Obese patients who received high-intensity diuretic therapy had an increased risk of deterioration of renal function within 72 h of treatment compared to a control group. Furthermore, high-intensity diuretic treatment was associated with a higher frequency of decline in renal function, which was similar in both obese and non-obese patients (3).

A recently described novel pathophysiological mechanism of muscle mass loss related to sodium metabolism in patients with chronic heart failure (CHF) involves active conservation of total body water during acute changes in sodium balance. This mechanism resembles the state of aestivation observed in animals for water conservation, resulting in metabolic regulation aimed at increasing the contribution of nonionic osmolytes, such as urea and glucose (4). In a study by Nihlén S. et al. (5), it was found that during treatment in the intensive care unit (ICU), diuretic-induced iatrogenic dehydration is associated with a shift toward the intensive production of organic osmolytes, mainly urea.” (Use “toward” instead of “towards” for consistency with American English). This adaptation is part of a universal response to water deficiency, observed in a wide range of organisms, from aestivating worms to higher animals, including mammals (6). This aligns with scientific data indicating that fluid loss can trigger protein breakdown in various cell types (7). Manifestations of aestivation in mammals include the synthesis of amino acid osmolytes, which contribute to increased plasma urea concentration, reduced urine output (oliguria), peripheral hypoperfusion, and muscle mass loss. The interplay of these mechanisms with varying levels of sodium intake in patients is intriguing (8), considering that individuals on a high-salt diet typically have significant depots of osmotically neutral sodium, including in muscle tissue (9, 10). These mechanisms may explain the high prevalence of sarcopenia in patients with CHF (11).

Importantly, patients with chronic HF often enter the hospital on chronically high salt diets; their interstitial tissues can store millimolar quantities of osmotically neutral Na^+^ (11). We hypothesized that intensive loop diuresis “unmasks” this hidden sodium, simultaneously triggering an Na^+^ → urea osmolyte switch and accelerating muscle loss—an effect that may be exaggerated when dietary salt is unrestricted.

Understanding this switch is clinically actionable for several reasons:

A rising urea-to-osmolality ratio during the first treatment week may signal that further loop-diuretic escalation will yield diminishing natriuretic returns while promoting catabolism; it can also help the clinician choose the optimal delivery strategy—high-dose intermittent boluses vs. lower-dose continuous (infusion) therapy—so that decongestion is achieved without exposing the patient to unnecessary metabolic stress. Because increased urea production often precedes measurable loss of lean body mass, tracking this ratio could identify individuals who would benefit from nutritional or exercise interventions. Furthermore, recognition of aestivation-like metabolic changes may guide clinical decision-making regarding the timing and intensity of diuretic therapy, particularly in patients with concurrent high sodium intake. Clinicians should consider monitoring the urea-to-osmolality ratio as a biomarker for early detection of catabolic stress, potentially prompting earlier implementation of protein supplementation, physical therapy interventions, or adjustment of diuretic dosing strategies to minimize muscle wasting while maintaining effective decongestion.

Objective of the Study: To evaluate the impact of varying levels of sodium intake and diuretic therapy on biochemical changes and the activation of aestivation mechanisms in patients with chronic heart failure (CHF).

## 2 Materials and methods

### 2.1 Study design

**Figure 1:**
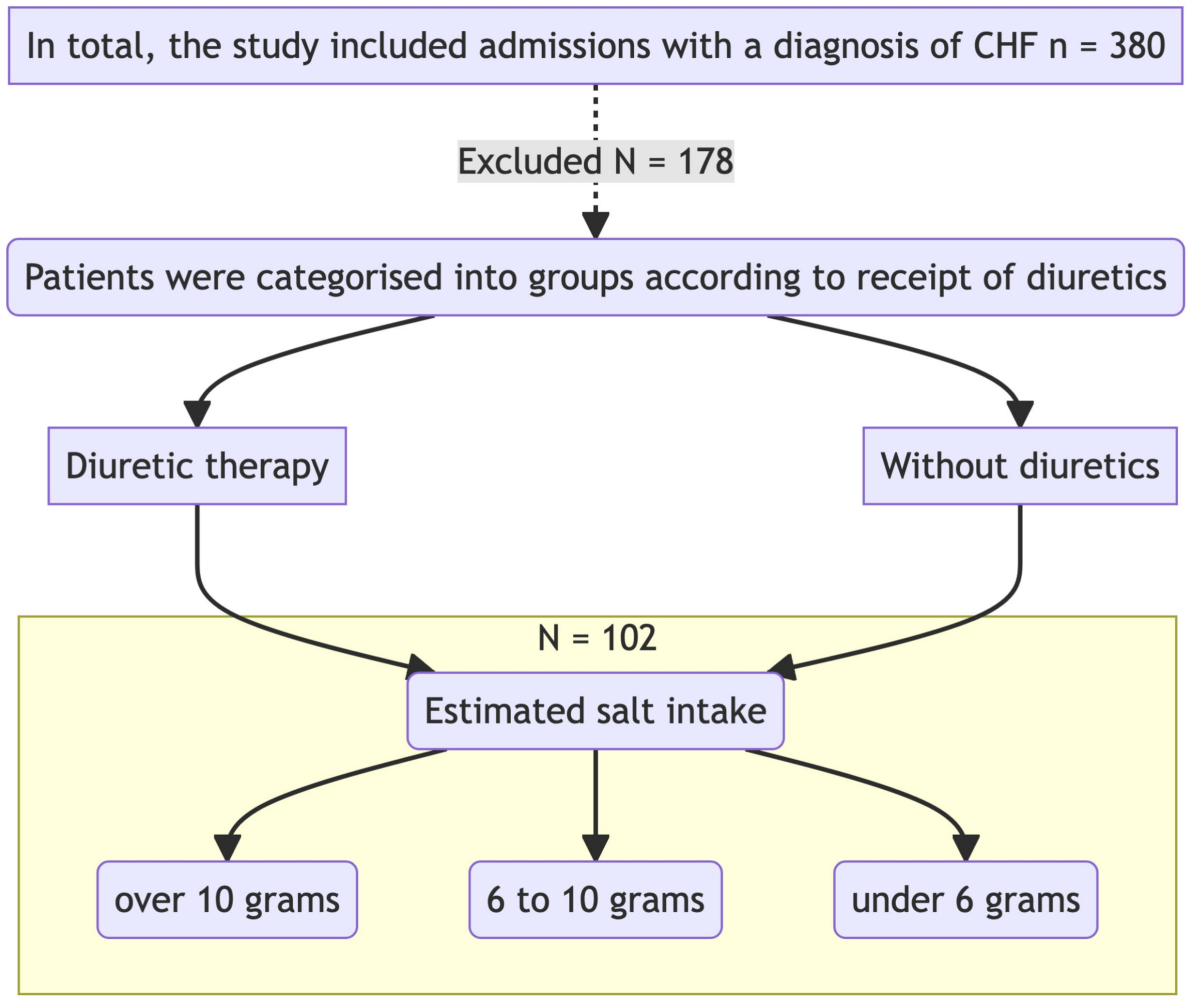
Research design.

The study was carried out in the Cardiology Department of GBUZ GVV No. 3 DZM, with patient recruitment taking place from January 2023 to July 2023. This was a prospective single-center open-label cohort study that aimed to investigate the impact of intensive diuretic therapy on the development of aestivation in patients with chronic heart failure (CHF) on diets with varying sodium content. The study adhered to the principles of the Helsinki Declaration. The study design is presented in Figure 1.

Upon admission, patients were divided into two groups according to the required level of diuretic therapy with loop diuretics. Biochemical analyses were performed on the first day of hospitalization before the initiation of diuretic therapy and after 7 days of therapy. The concentrations of sodium, potassium, glucose, urea, albumin, total protein, aspartate aminotransferase (AST), alanine aminotransferase (ALT), creatine kinase (CK), high-density lipoprotein (HDL), low-density lipoprotein (LDL), triglycerides, cholesterol, creatinine, and C-reactive protein (CRP) were measured.

### 2.2 Inclusion and exclusion criteria

Participants: Patients with a confirmed diagnosis of chronic heart failure, who had been on stable therapy (ACE inhibitors/ARBs, beta-blockers at more than 50% of the maximum dose) for more than 3 weeks, were included in the study. According to current clinical guidelines and evidence from major randomized trials, a dose of ≥50% is necessary to achieve the proven clinical benefits of beta-blocker therapy, including reduced mortality and hospitalization. Patients receiving lower doses may represent a less stable population (e.g., undergoing dose titration or with poor tolerance), which could introduce confounding variability in the assessment of metabolic and osmotic adaptations. None of the patients were receiving neprilysin/angiotensin receptor inhibitors (ARNI, such as sacubitril/valsartan) or sodium-glucose co-transporter 2 inhibitors (SGLT2 inhibitors, such as empagliflozin or dapagliflozin) at the time of inclusion in the study. This was due to the limited availability of these medications in the inpatient setting during the study period and their absence from standard therapy regimens for the majority of patients in the observed cohort. The main inclusion and exclusion criteria are presented in Table 1. The study was conducted in accordance with the Helsinki Declaration and approved by the ethics committee.

**Table 1:** Inclusion and exclusion criteria.

Inclusion criteria	Exclusion criteria
Presence of an established diagnosis of CHF (NYHA I- IV Functional class)	Unlikely cooperation with the patient during the trial, low adherence to therapy for social, psychological, economic, and other reasons, incapacity
Stable therapy for 3 months prior to inclusion in the study	Patients with cancer who are on radiation therapy or chemotherapy
Stable therapy with ACE inhibitors/ARBs, beta-blockers for 3 weeks prior to hospitalization	Need to be in the intensive care unit at the time of admission
Signing voluntary informed consent	Presence of any severe, decompensated, or unstable chronic somatic disease that, in the opinion of the investigators, could affect study outcomes or patient safety. These included, but were not limited to: Severe anemia (hemoglobin < 90 g/L) Decompensated or poorly controlled endocrine disorders (e.g., uncontrolled diabetes mellitus with HbA1c > 9%, untreated hypothyroidism or hyperthyroidism, adrenal insufficiency) Systemic autoimmune diseases (e.g., systemic lupus erythematosus, vasculitis, rheumatoid arthritis with high inflammatory activity) Advanced liver disease (e.g., liver cirrhosis Child-Pugh class B or C, active hepatitis) Severe chronic pulmonary diseases (e.g., COPD with frequent exacerbations or requiring oxygen therapy) Advanced neurological disorders impairing mobility or cognitive function (e.g., Parkinson’s disease, recent stroke with residual deficit)
Constant salt intake according to the food diary during the week before hospitalization	Abuse of alcohol, drugs, or medicines
No loop diuretic therapy prior to inclusion in the study for 2 weeks	Taking thiazide or thiazide-like diuretics

### 2.3 Laboratory analyses

Biochemical parameters—including sodium, potassium, glucose, urea, creatinine, total protein, albumin, aspartate aminotransferase (AST), alanine aminotransferase (ALT), creatine kinase (CK), total cholesterol, triglycerides, high-density lipoprotein (HDL), low-density lipoprotein (LDL), and C-reactive protein (CRP)—were measured using standard laboratory methods in the hospital’s certified clinical diagnostic laboratory.

The following equipment was used:

- Beckman Coulter DxC700 AU (Ireland)—for biochemical analysis
- CL-2000i (Mindray, China)—chemiluminescent immunoassay analyzer
- Cobas e 411 (Roche Diagnostics, Japan)—immunoassay analyzer
- XN-Series (Sysmex, Japan)—for hematology analysis

CRP levels were assessed using an immunoturbidimetric assay. All laboratory procedures were performed according to the manufacturers’ protocols with proper calibration and internal quality control procedures in place.

### 2.4 Calculation of plasma Osmolarity


(1) $$\text{eOSM}=2*Na^{+}+2*K^{+}+U+\mathrm{Gl}$$

In Equation 1, where $U_{n}$ is plasma urea, $Gl_{n}$ is plasma glucose, and eOSM is the estimated plasma osmolality.

Plasma osmolality was assessed according to Equation 1. We then calculated the proportions of each osmolyte relative to the estimated plasma osmolality, obtaining the following ratios: Prop _Na/eOSM_, Prop _K/eOSM_, Prop _Urea/eOSM_, and Prop _Glucose/eOSM_, respectively.

### 2.5 Methods for determining salt intake

Salt intake was evaluated using a diet questionnaire in which patients recorded their dietary information for any two weekdays and one weekend day during the week prior to hospitalization. The electronic questionnaire was published on the website http://www.saltquest.ru/Sodium_project/. The electronic questionnaire contained a pre-formed database of foods produced in Russia with known sodium content per 100 grams of product or dish. All individual products and recipes were grouped into similar types of food (e.g., popcorn, potato chips, crackers) and then combined into broader categories (e.g., snacks), which formed the foundation of the food diary.

When filling out the food diary, the type of meal (breakfast, lunch, dinner), its volume, and the fact that the dish was additionally salted were taken into account. The volume of additional salting was calculated based on 0.1 grams of salt per additional salting.

Patients with a consistent level of sodium intake were included in the study. Weekly intake fluctuations were allowed within ±2 grams per day, provided that they did not exceed the salt intake categories: (1) less than 6 grams, (2) 6 to 10 grams, (3) more than 10 grams per day (12).

### 2.6 Definition of heart failure

The presence of heart failure in patients was determined based on the National Clinical Guidelines for the Diagnosis and Treatment of Chronic Heart Failure (CHF) (13). The stage and functional class of CHF for each patient included in the final analysis were determined by two independent experienced cardiologists. If their assessments differed, the final decision was made after joint discussion.

Ejection fraction (EF) for all patients was determined using the Simpson’s method in the apical four-chamber and two-chamber views, and the average EF was calculated:

- Heart failure with preserved ejection fraction (HFpEF): EF ≥ 50%
- Heart failure with mildly reduced ejection fraction (HFmrEF): EF 41–49%
- Heart failure with reduced ejection fraction (HFrEF): EF ≤ 40%

These thresholds were used in combination with clinical signs and symptoms, natriuretic peptide levels, and echocardiographic parameters (such as left atrial volume index and left ventricular mass index), as recommended in the guidelines.

Lifestyle characteristics were assessed at baseline using a structured questionnaire. The questionnaire included information on:

- Physical activity: categorized as low (sedentary), moderate (regular walking or light activity), or high (structured exercise ≥3 times/week);
- Smoking status: current smoker, former smoker, or never smoked;
- Alcohol consumption: categorized as none, occasional (≤2 times/week), or regular (>2 times/week)

### 2.7 Determination of kidney function and verification of chronic kidney disease

The glomerular filtration rate (GFR) was calculated using the CKD-EPI (Chronic Kidney Disease Epidemiology Collaboration) 2011 formula (14), according to the National Guidelines for Chronic Kidney Disease (CKD). Albuminuria was determined using test strips and the albumin/creatinine ratio was measured in a morning urine sample. The diagnosis of CKD was verified according to the guidelines (15), based on the following criteria:

- The presence of any clinical markers of kidney damage was confirmed twice, with a time interval of at least 3 months between tests.
- Detected decrease in GFR (>60 mL/min/1.73 m^2^), albuminuria, or any other clinical markers of kidney damage confirmed over a period of 3 months.
- Persistent GFR < 60 mL/min/1.73 m^2^ regardless of the dynamics of other markers.
- Diagnosis of irreversible markers (signs) of structural kidney changes confirmed by biopsy or imaging studies.

### 2.8 Sample size calculation

The sample size for the study was calculated based on the results of the study by Nihlen et al. (5). We assumed that the expected mean difference between the diuretic groups receiving and those not receiving them would be around 4% in the proportion of urea, with a standard deviation not exceeding 5%. Given the limited number of studies that have investigated changes in urea levels during diuretic therapy, a coefficient of variation level of 10% was adopted. This provided sufficient confidence that the calculated confidence limits would be reliable enough to confirm the study results at a power of 90%. This corresponded to a minimum sample size of 34 patients to assess the possible change in urea levels during diuretic therapy.

Taking into account the potential exclusion of respondents due to incomplete adherence to the study protocol and the possible reduction in the representative sample after the analysis of propensity matching, the sample size was increased by 50–70% from the calculated size. Therefore, the required sample size was determined to be at least 51 individuals. This number was sufficient to obtain statistically significant results. Therefore, an adequate sample size was formed to carry out the study.

### 2.9 Statistical analysis

Statistical analysis of the data obtained was performed using R, version 4.3.2, in the RStudio development environment (packages: ggplot2, ggpubr, dplyr, tidyverse, gtsummary, rstatix). The normality of the distribution was determined using the Shapiro–Wilk test, as well as the Kolmogorov–Smirnov test. We also examined the values of skewness and kurtosis and constructed QQ plots and distribution histograms.

Quantitative data were presented as mean (M) ± standard deviation (SD) or median with the 25th and 75th percentiles. Both parametric and nonparametric statistical methods were used to describe the results. The Kruskal-Wallis test or analysis of variance (ANOVA) was used to compare multiple groups. To compare two groups, Student’s *t*-test was applied for normally distributed data and the Wilcoxon rank sum test was used for non-normally distributed data.

For categorical variables, frequency tables were constructed and checked using the Chi-square test with Yates correction. Fisher’s exact test was used when the group size was less than 5, followed by *post hoc* analysis with Holm’s correction for multiple comparisons. The Spearman correlation coefficient was used to study the relationship between variables. Logistic regression (both univariate and multivariate) was applied to examine the association between categorical dependent variables with multiple categories.

To adjust for predefined confounding factors in our analysis, we used the matching of propensity score (16). The cohort of interest was matched using the MatchIt library (17) with the ‘full’ matching method, which provided the best match based on standardized mean differences. The matching model included the following covariates: Age, Left ventricular ejection fraction (EF), Estimated glomerular filtration rate (eGFR).

Missing data handling: Only patients with complete data for both day 1 and day 7 laboratory assessments were included in the main comparative analyses. Cases with missing values for key variables (electrolytes, urea, glucose, eGFR) were excluded from the respective comparisons. No imputation techniques were applied.

Statistical hypotheses were tested with the null hypothesis rejected at a significance level of less than 0.05.

## 3 Results

### 3.1 Clinical characteristics of the group

A total of 102 individuals were included in the study, with a median age of 75 years (minimum age 43, maximum age 93 years). The number of women was slightly higher than that of men: 59 (57.8%).

The predominant etiologies of chronic heart failure (CHF) among enrolled patients were ischemic heart disease, including a history of myocardial infarction (67%, *n* = 68), and arterial hypertension without significant coronary artery disease (33%, *n* = 34). Other causes of CHF were not represented in the study cohort. The clinical characteristics of each group, based on whether they received diuretic therapy, are presented in Table 2. For most parameters, the patients did not differ significantly. However, significant differences were observed in some parameters; for example, the patients in the diuretic therapy group were older than those in the non-diuretic therapy group. Patients requiring diuretics generally had higher stages and functional classes of CHF. Consequently, this group had more patients with lower ejection fractions, and due to the interrelationship between CHF and chronic kidney disease (CKD), patients in the diuretic group often had higher stages of CKD.

**Table 2:** Clinical characteristics of patients in the group who received diuretics and those who did not received diuretics.

Characteristic	Patients receiving diuretics, *N* = 59^1^	Patients not receiving diuretics, *N* = 43^1^	*p*-value^2^
Age in years, median [Q25; Q75]	81 (72, 84)	73 (67, 79)	0.011
Gender			>0.9
Female (%)	34 (58%)	25 (58%)	
Male (%)	25 (42%)	18 (42%)	
Sodium 1 day (mMol/L)	138.7 ± 3.1	138.6 ± 4.3	>0.9
Potassium 1 day (mMol/L)	4.07 ± 0.43	4.00 ± 0.41	0.3
Urea 1 day (mMol/L)	8.4 ± 4.3	7.1 ± 2.6	0.15
Glucose 1 day (mMol/L)	6.58 ± 2.21	6.46 ± 1.68	0.7
Sodium day 7 (mMol/L)	139.32 ± 2.64	139.51 ± 3.78	0.9
Potassium day 7 (mMol/L)	4.34 ± 0.53	4.15 ± 0.47	0.042
Urea day 7 (mMol/L)	9.2 ± 4.1	6.4 ± 1.8	<0.001
Glucose day 7 (mMol/L)	6.04 ± 1.66	5.75 ± 1.39	0.5
Functional class of CHF (NYHA)			<0.001
NYHA I	5 (8.5%)	18 (42%)	
NYHA II	27 (46%)	25 (58%)	
NYHA III	26 (44%)	0 (0%)	
NYHA IV	1 (1.7%)	0 (0%)	
Ejection fraction (%)	48 ± 8	51 ± 4	0.049
Estimated osmolarity on day 1 (mOsm/L)	301 ± 8	299 ± 9	0.5
Estimated osmolarity on day 7 (mOsm/L)	302.5 ± 6.8	299.5 ± 7.8	0.039
Estimated glomerular filtration rate (ml/min/1.73 m2)	50 ± 15	57 ± 14	0.021
Albumin (g/l)	39.2 (37.1, 40.8)	39.2 (37.0, 41.4)	0.8
Total protein (g/l)	67.8 (65.1, 71.0)	68.5 (64.8, 71.5)	>0.9
ALT (U/L)	18 (12, 29)	17 (13, 23)	0.5
AST (U/L)	20 (16, 29)	20 (18, 25)	0.8
HDL (mMol/L)	1.31 (1.17, 1.45)	1.26 (1.06, 1.34)	0.2
LDL (mMol/L)	2.80 (2.18, 3.29)	2.63 (2.20, 3.27)	>0.9
Triglycerides (mMol/L)	1.30 (0.91, 1.71)	1.27 (0.85, 1.84)	0.9
Cholesterol (mMol/L)	4.50 (3.64, 5.40)	4.60 (3.50, 5.45)	>0.9
CRP (mg/L)	5 (3, 10)	5 (2, 8)	0.3
Diabetes mellitus			0.7
No (%)	39 (66%)	30 (70%)	
Yes (%)	20 (34%)	13 (30%)	
CKD (stage)			0.025
C1	0 (0%)	1 (2.3%)	
C2	1 (1.7%)	5 (12%)	
C3a	24 (41%)	23 (53%)	
C3b	16 (27%)	4 (9.3%)	
C4	5 (8.5%)	1 (2.3%)	
No	13 (22%)	9 (21%)	
Physical activity: Low (%)	26 (44%)	18 (42%)	0.8
Physical activity: Moderate (%)	24 (41%)	17 (40%)	0.6
Physical activity: High (%)	9 (15%)	8 (18%)	0.7
Smoking status: Current smoker (%)	6 (10%)	5 (12%)	0.7
Smoking status: Former smoker (%)	18 (31%)	16 (37%)	0.9
Smoking status: Never smoked (%)	30 (50%)	18 (42%)	0.9
Alcohol consumption: None (%)	16 (27%)	14 (33%)	0.6
Alcohol consumption: Occasional (%)	13 (22%)	11 (26%)	0.7

1 Median (IQR); n (%).

2 Wilcoxon rank sum test; Pearson’s Chi-squared test; Fisher’s exact test.

The average doses of diuretics during the 7-day observation period were as follows: furosemide – 39.1 ± 22.1 mg, torasemide—7.4 ± 3 mg, and spironolactone—42 ± 12.4 mg. Patients receiving thiazide or thiazide-like diuretics were not included in the study.

The diuretic dosages for patients with different stages of CKD were distributed as follows: for CKD stage 3a, the average dose of furosemide was 34.0 mg ± 13.5 mg, torasemide—7.3 mg ± 2.9 mg, and spironolactone—37.5 mg ± 14.4 mg. For CKD stage 3b, the average dose of furosemide was 40.0 mg ± 16.3 mg, torasemide—7.3 mg ± 3.0 mg, and spironolactone—35.7 mg ± 13.4 mg. In patients with CKD stage 4, the average dose of furosemide increased to 60.0 mg ± 52.9 mg.

On the first day of observation, the mean estimated osmolality in the group of patients receiving treatment (*n* = 59) was 300 [297; 304] mOsm/L, which was comparable to the group of patients not receiving treatment (*n* = 43), where the value was 299 [296; 302] mOsm/L with a *p*-value > 0.5, indicating that there was no statistically significant difference. However, on the seventh day, a statistically significant increase in osmolality was observed in the treatment group, reaching 302.2 [298.3; 305.8] mOsm/L compared to the nontreatment group, which had a value of 300.2 [295.9; 303.5] mOsm/L, with a p-value of 0.039. These data highlight the potential impact of therapeutic interventions on plasma osmolality as part of the treatment approach for chronic heart failure.

### 3.2 Changes in plasma osmolyte ratios on day 7 of diuretic therapy

**Figure 2:**
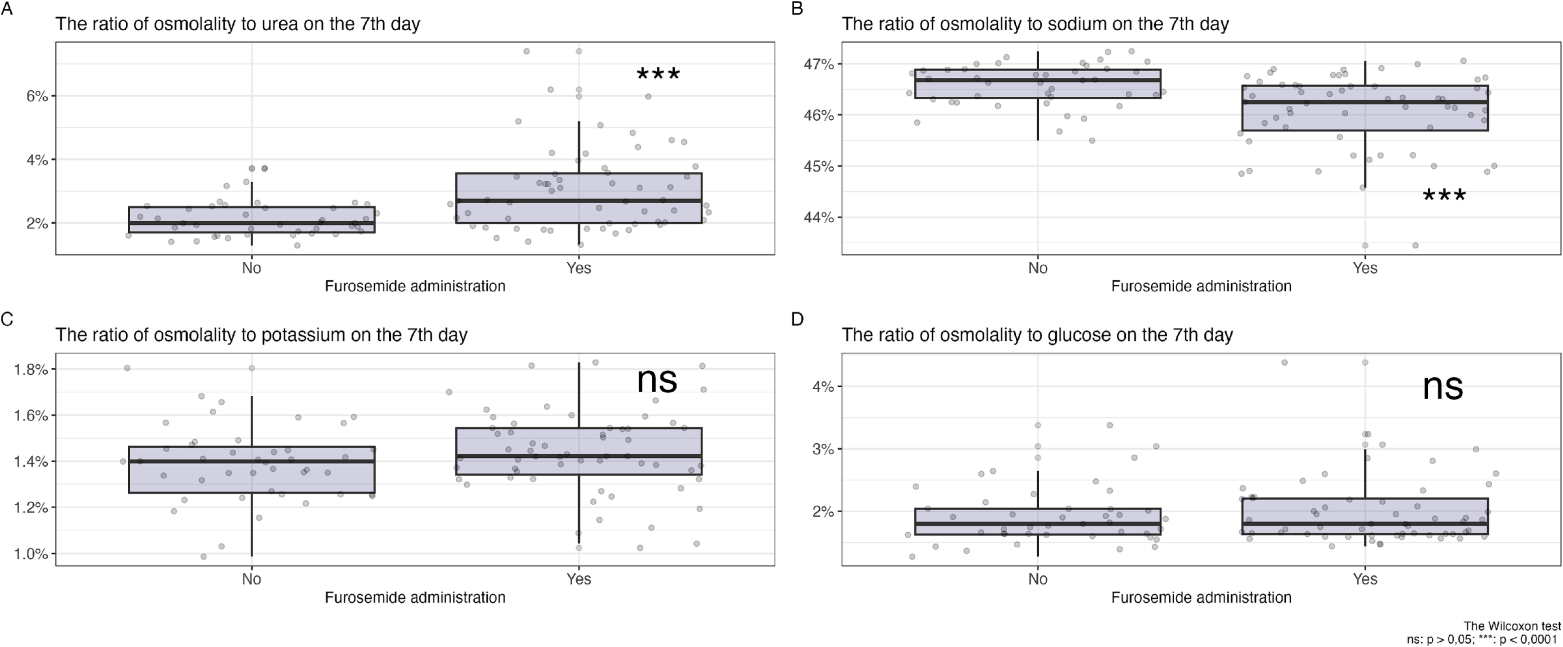
Changes in the ratio of osmolytes in plasma on the 7th day of diuretic therapy are detailed as follows: **(А)** osmolarity to urea ratio, **(B)** osmolarity to sodium ratio, **(С)** osmolarity to potassium ratio, **(D)** osmolarity to glucose ratio.

On the first day (Figure 2) of observation, for patients who did not receive diuretics, no statistically significant differences were identified in the proportions of osmolytes relative to plasma osmolality between the groups planned for diuretic treatment and those who did not receive such treatment (glucose 2[1.8; 2.3] vs. 2[1.8; 2.4] *p* = 0.66, potassium 1.3[1.3; 1.4] vs. 1.3[1.3; 1.4] *p* = 0.35, sodium 46.3[45.9; 46.7] vs. 46.4[46.1; 46.8] *p* = 0,14, urea 2.5[1.9; 3.4] vs. 2.2[1.8; 2.6] *p* = 0.15).

In Figure 2, a comparison of the changes in the proportions of key plasma osmolytes is shown after 7 days of loop diuretic treatment. Changes in potassium and glucose levels did not show statistically significant differences, while an increase in the proportion of urea and a decrease in the proportion of sodium in plasma osmolality were observed in the group receiving diuretic therapy.

Therefore, after 7 days of diuretic treatment, a statistically significant increase in the proportion of urea and a decrease in the proportion of sodium were observed.

### 3.3 Relationship between plasma osmolytes and sodium intake levels

**Figure 3:**
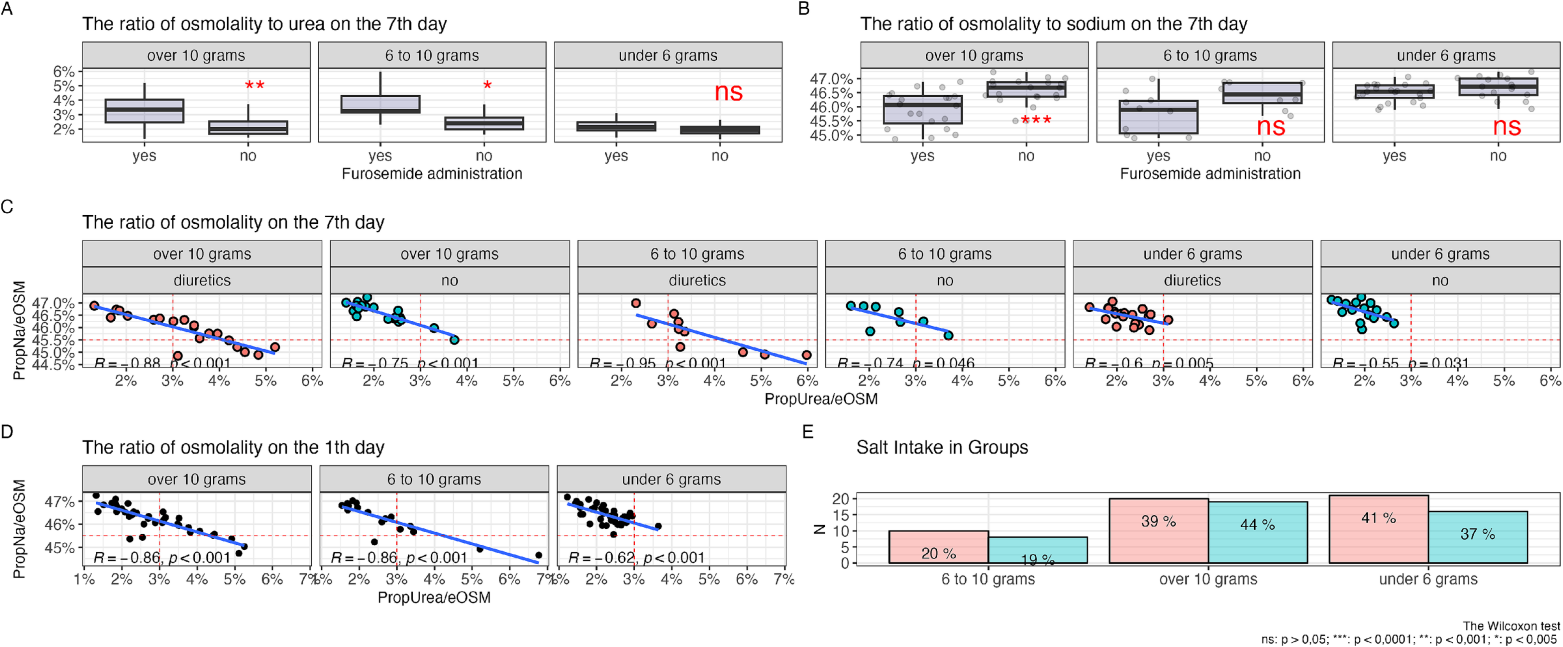
Relationship of osmolytes with the level of salt intake, **(А)** ratio of urea to plasma osmolarity among patients with varying levels of salt intake, **(B)** ratio of sodium to plasma osmolarity among patients with varying levels of salt intake, **(С)** correlation of the ratios of urea and sodium to plasma osmolarity among patients with different levels of salt intake undergoing diuretic therapy over 7 days, **(D)** correlation of the ratios of urea and sodium to plasma osmolarity among patients with different levels of salt intake on the first day of the study without diuretics, **(E)** salt intake in the diuretic therapy group (red column) and without diuretics (green column).

In Figure 3A, it is shown that during diuretic therapy, patients who consumed 10 grams of salt per day experienced a statistically significant increase in the proportion of urea compared to other osmolytes. A similar effect was observed with a diet of 6 to 10 grams of salt per day. Meanwhile, on a low-salt diet, no statistically significant relationship was observed. PropNa/eOSM (Figure 3B) showed statistically significant changes only with a diet of 10 grams of salt per day; with a salt intake of 6 to 10 grams per day, a similar trend was observed, but it was statistically non-significant, and no changes were observed with a diet of less than 6 grams per day.

Figure 3C shows a negative linear relationship between the proportions of urea and sodium relative to other osmolytes, with the most pronounced correlation seen with diets consuming 10 grams and 6 to 10 grams of salt per day during diuretic therapy. Furthermore, there was a shift in the proportion of urea to values above 3% and a decrease in the proportion of sodium below 45.5%, while without diuretics, this observation was not present in diets of 10 grams and 6–10 grams of salt per day, and the correlation level remained almost the same. With a salt intake of less than 6 grams per day, the linear relationship was less pronounced and almost all observations were concentrated in the range with a proportion of urea less than 3% and a proportion of sodium more than 45.5%.

Figure 3D demonstrates a negative linear relationship between the proportions of urea and sodium relative to other osmolytes before the start of diuretic therapy, with a more pronounced negative linear trend with diets of 10 grams and 6 to 10 grams of salt per day, and a less pronounced trend with a diet of less than 6 grams of salt per day.

In Figure 3E, the level of salt intake among groups of patients receiving diuretic therapy and those not receiving it is shown. The largest number of patients were observed in the groups with a diet that included a salt intake of 10 grams and less than 6 grams per day, and to a lesser extent in the 6–10 grams per day group. There were no statistically significant differences between the groups (*p* = 0.6).

### 3.4 Propensity score matching

Taking into account the presence of statistically significant differences between the compared groups (with and without diuretics) in terms of age, CHF stage, ejection fraction, glomerular filtration rate (GFR) and chronic kidney disease (CKD), pseudorandomisation was performed using the propensity score matching (PSM) method to eliminate the potential influence of CHF severity or significant contributions from renal disease and function in patients. After the pseudorandomisation procedure, the total number of patients was reduced to 71. However, biases in the aforementioned parameters were eliminated and the results are presented in Table 3.

**Table 3:** Group differences in key clinical characteristics after adjustment using propensity score matching (PSM).

Characteristic	Patients receiving diuretics, *N* = 31^1^	Patients not receiving diuretics, *N* = 43^1^	*p*-value^2^
Age (years)	73 (65, 77)	73 (67, 79)	0.6
Ejection fraction (%)	52.0 (50.0, 53.0)	52.0 (50.0, 52.0)	0.8
Estimated glomerular filtration rate (ml/min/1.73 m2)	55 (49, 66)	56 (48, 65)	0.7
CKD (stage)			0.2
C1	0 (0%)	1 (2.3%)	
C2	1 (3.2%)	5 (12%)	
C3a	13 (42%)	23 (53%)	
C3b	4 (13%)	4 (9.3%)	
C4	0 (0%)	1 (2.3%)	
нет	13 (42%)	9 (21%)	

1 Median (IQR); n (%).

2 Wilcoxon rank sum test; Pearson’s Chi-squared test; Fisher’s exact test.

In Table 4, the results of the impact of various osmolytes on osmolality after diuretics application, performed following the propensity score matching procedure, are presented. The table indicates that despite the balance of the sample for renal function, there are still statistically significant differences in the levels of urea, PropUrea/eOSM, and PropNa/eOSM on the seventh day of diuretic therapy. These changes suggest that the alterations in the contribution of urea to osmolality are not related to changes in renal function or a higher degree of CHF in patients, but are directly caused by the impact of diuretic therapy on muscle tissue, leading to increased urea synthesis and a reduction in the sodium proportion.

**Table 4:** Relationship of plasma osmolyte concentrations and their ratios to estimated plasma osmolality (eOSM) in patients receiving and not receiving diuretics after adjustment using propensity score matching (PSM).

Characteristic	Patients receiving diuretics, *N* = 28^1^	Patients not receiving diuretics, *N* = 43^1^	*p*-value^2^
Sodium day 1 (mMol/L)	138.90 (137.33, 140.53)	138.80 (137.20, 140.70)	>0.9
Potassium day 1 (mMol/L)	3.97 ± 0.38	4.00 ± 0.41	>0.9
Glucose day 1 (mMol/L)	5.81 (5.40, 6.67)	5.91 (5.39, 7.09)	0.6
Urea day 1 (mMol/L)	6.45 (5.15, 8.10)	6.60 (5.50, 7.95)	0.6
Sodium day 7 (mMol/L)	139.40 (138.58, 140.93)	139.80 (138.45, 141.20)	0.8
Potassium day 7 (mMol/L)	4.17 ± 0.48	4.15 ± 0.47	0.7
Glucose day 7 (mMol/L)	5.26 (4.78, 6.52)	5.33 (4.87, 6.25)	0.9
Urea day 7 (mMol/L)	7.95 (5.65, 9.90)	5.90 (5.05, 7.50)	0.012
Urea to osmolarity ratio (%)	2.63 (1.89, 3.28)	2.00 (1.70, 2.50)	0.011
Sodium to osmolarity ratio (%)	46.46 (46.02, 46.74)	46.68 (46.33, 46.89)	0.050
Potassium to osmolarity ratio (%)	1.40 (1.32, 1.49)	1.40 (1.26, 1.46)	0.8
Glucose to osmolarity ratio (%)	1.74 (1.59, 2.19)	1.80 (1.63, 2.04)	0.9

1 Median (IQR).

2 Wilcoxon rank sum test; Wilcoxon rank sum exact test.

**Figure 4:**
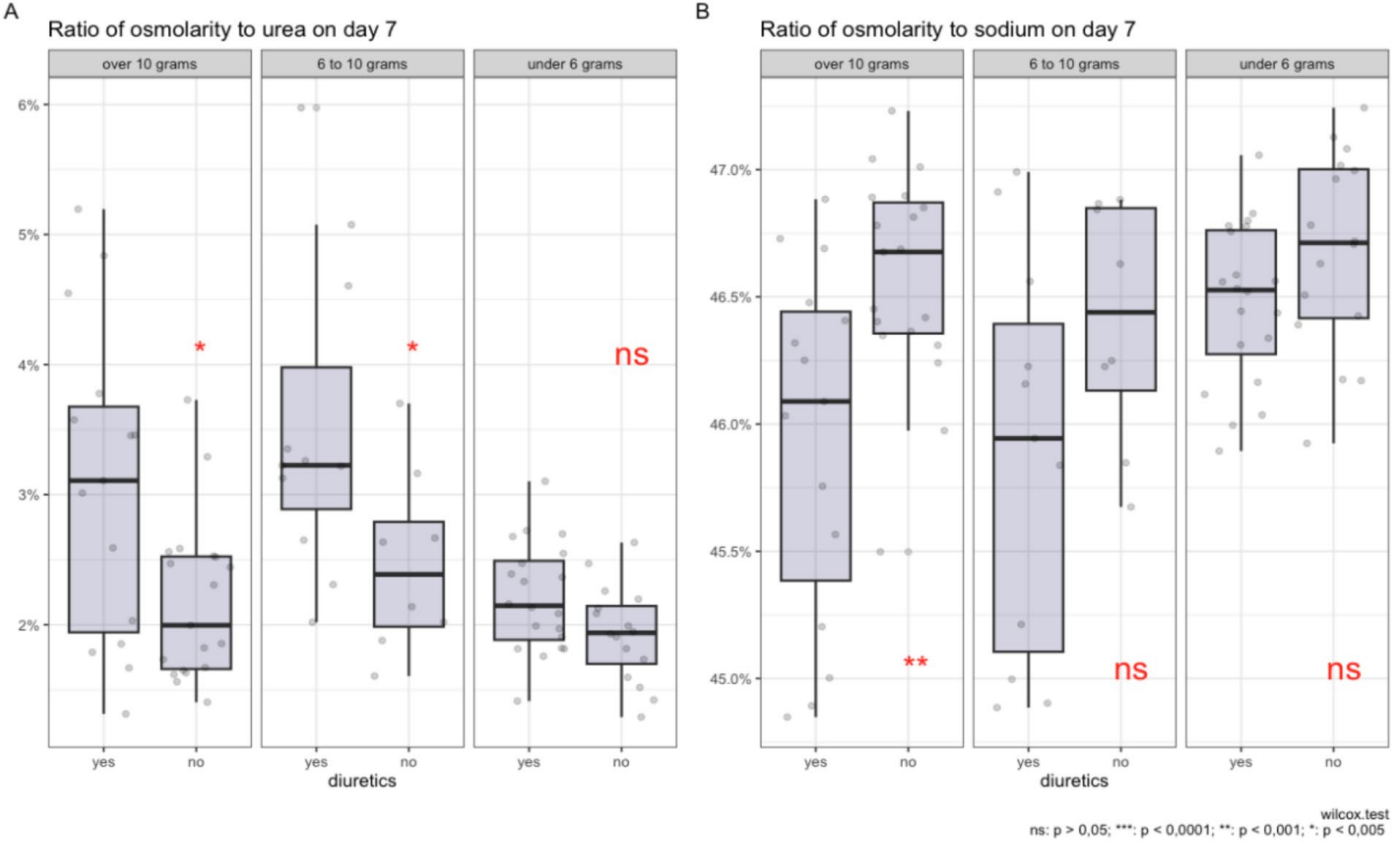
Comparison of the ratio of plasma osmolality to urea **(A)** and sodium **(B)** on day 7 of diuretic therapy across groups with varying levels of salt intake, after data adjustment using propensity score matching.

In Figures 4A,B, the impact of salt intake and diuretic therapy on the contribution of key electrolytes to plasma osmolality is illustrated, following the PSM procedure.

In graph A, it is shown that with high salt intake (more than 10 grams and from 6 to 10 grams per day), the use of diuretics is associated with a higher percentage contribution of urea to the total plasma osmolality. For salt intake of less than 6 grams per day, there is no statistically significant increase in the contribution of urea.

In graph B, a significant reduction in the sodium proportion in plasma osmolality is observed with a salt intake of more than 10 grams per day during diuretic therapy. Although statistical significance is not reached for the 6–10 grams range and less than 6 grams of salt per day, a trend toward a reduction in the proportion of sodium is still observed for the 6–10 gram group. The contribution of potassium and glucose to osmolality remains statistically insignificant regardless of the level of salt intake and diuretic use.

The box plots illustrate the distribution of median values, interquartile ranges, and individual measurements, providing a visual representation of changes in plasma electrolyte composition under the influence of these factors, adjusted using the PSM method.

### 3.5 Modeling results

In Table 5, the results of the modeling of the use of diuretics against the background of a diet with varying levels of sodium intake and the changes in the contributions of various osmolytes to plasma osmolality, such as urea, glucose, sodium, and potassium, and their impact on the likelihood of diuretic use in patients are presented.

**Table 5:** Multivariate logistic regression models evaluating the association between the use of diuretics and plasma osmolyte ratios on day 7, with adjustment for salt intake.

Переменные	Model 1			Model 2			Model 3			Model 4		
	OR^1^	95% CI^1^	*p*-value	OR^1^	95% CI^1^	*p*-value	OR^1^	95% CI^1^	*p*-value	OR^1^	95% CI^1^	*p*-value
PropK/eOSM	6.45	0.62, 78.3	0.12									
Salt intake			0.5			0.6			0.2			0.3
Over 10 grams	—	—		—	—		—	—		—	—	
6 to 10 grams	1.68	0.68, 4.20		1.51	0.62, 3.71		2.65	0.97, 7.71		2.29	0.86, 6.39	
Under 6 grams	1.70	0.57, 5.30		1.54	0.53, 4.69		1.39	0.40, 4.88		1.54	0.46, 5.38	
PropGlucose/eOSM.				1.32	0.60, 3.13	0.5						
PropUrea/eOSM							3.52	1.94, 7.26	**<0.001**			
PropNa/eOSM										0.16	0.06, 0.39	**<0.001**
AIC	143			145			124			126		

1 OR, Odds Ratio; CI, Confidence Interval. Bold values indicate *p* < 0.001.

Model 1: Analysis of PropK/eOSM on the seventh day and various levels of salt intake yielded an odds ratio (OR) of 6.45 with a 95% confidence interval (CI) of [0.62; 78.3] and a *p*-value of 0.12. This indicates that there is no statistically significant effect of osmolality relative to potassium on the likelihood of diuretic use.

Model 2: Evaluation of PropGlucose/eOSM on day 7 and various levels of salt intake showed an OR of 1.32 with a 95% CI of [0.60; 3.13] and a *p*-value of < 0.5. Here, too, no statistically significant results were obtained, indicating that there was no dependence between diuretic use and changes in PropGlucose/eOSM.

Models 3 and 4: These models provide estimates for PropUrea/eOSM and PropNa/eOSM on the seventh day. In Model 4, the OR estimate for PropUrea/eOSM on day 7 is 3.52 with a 95% CI of [1.94; 7.26] and a *p*-value of < 0.001. This indicates a significant association between diuretic use and changes in PropUrea/eOSM. In contrast, an increase in osmolality compared to sodium on day 7 is associated with a significant reduction in the likelihood of diuretic use (OR = 0.16, 95% CI: 0.06–0.39, *p* < 0.001).

Thus, the results confirm that the use of diuretics in combination with a diet containing varying amounts of salt induces different changes in PropUrea/eOSM and PropNa/eOSM.

When constructing univariate models that evaluate the use of diuretics, PropNa/eOSM on day 7 and PropUrea/eOSM on day 7 without considering salt intake, statistically significant results are obtained. However, they are less robust compared to models that include the level of salt intake. For example, in the PropUrea / eOSM model, the odds ratio (OR) is 3.01 (95% CI 1.74, 5.89; *p* < 0.001), and for the PropNa / eOSM model, the OR is 0.19 (95% CI 0.07, 0.43; *p* < 0.001).

## 4 Discussion

The main finding of the present study is that patients with chronic heart failure (CHF) exhibit metabolic features resembling aestivation in response to loop diuretic therapy, particularly under conditions of high dietary sodium intake. This adaptation is characterized by a shift in plasma osmolyte composition—namely, an increase in the contribution of urea and a relative decrease in sodium—to maintain plasma osmolality and promote water conservation. The rise in the urea-to-osmolality ratio suggests enhanced proteolysis and nitrogen conservation, potentially indicating muscle catabolism. These mechanisms may contribute to the development of sarcopenia and reduced functional status commonly observed in CHF.

This study expands upon previously published findings in several important ways. It is the first clinical investigation to demonstrate a relationship between excessive sodium intake and the use of diuretics in patients with chronic heart failure, leading to dehydration. The results are consistent with prior studies conducted in cohorts of critically ill patients in intensive care units receiving diuretic therapy (5), where similar shifts in osmolyte composition—primarily an increased contribution of urea—were observed by day seven. These findings support the generalizability of the concept of metabolic aestivation as a universal adaptive response occurring under conditions of critical illness and fluid loss–associated therapy.

Aestivation can be characterized as a series of physiological adaptations aimed at preventing dehydration and death. The key goal of aestivation is the conservation of water in the body. The need to conserve water under conditions of high salt intake induces a state similar to aestivation in experimental mice (18).

Homer Smith was the first to study changes in osmolytes and body hydration in aestivating ‘lungfish’ (19). These fish, living in underground mud cocoons, enter a state of aestivation when in water that is hyperosmotic relative to their body fluids, which promotes water loss. To prevent dehydration, aestivating lungfish increase the level of urea in their plasma and tissues.

Urea and its transporters play a crucial role in the urine concentration processes in the kidneys. Studies show that in cases of protein deficiency, the ability to concentrate urine decreases, which can be restored by adding urea. Genetically modified mice lacking specific urea transporters demonstrate similar reductions in urine concentration capacity (18).

There is a hypothesis that a high concentration of urea in the interstitial space of the renal medulla is necessary for effective urine concentration. This is achieved by reabsorption of urea through specific protein transporters, such as UT-A1 and UT-A3. The UT-B1 transporter, present in erythrocytes, also plays an important role in this process, facilitating efficient countercurrent exchange and urine concentration. The absence of this transporter in humans and mice leads to a reduced urine concentration capacity (18).

The principle of water conservation in urine concentration through accumulation of urea has been previously established and confirmed in mice with impaired renal urea transporter function (20). According to previous data that indicate opposing effects of urea and NaCl osmolytes on urea transport managed by the UT-A1 transporter under conditions of acute osmotic diuresis, it is demonstrated that this principle of water conservation, dependent on urea, is used to maintain kidney concentration processes, possibly compensating for the osmotic diuretic effect of salt excretion. As a result, endogenous water accumulation ensures consistent urine volume despite intense natriuresis (20). These mechanisms are particularly relevant in patients with chronic heart failure undergoing diuretic therapy. The present study offers a novel perspective on intensive diuretic treatment, suggesting that it may activate distinct metabolic effects associated with the aestivation response.

Intense natriuresis can occur for various reasons and can be related to external factors that affect patients or the use of medications that increase natriuresis. Bankir et al. (21) previously noted that during the study ‘Dietary approaches to stop hypertension (DASH)’, increased salt excretion led to higher $Na^{+}$ concentrations in patient urine without increasing urine volume. These results underscore the importance of controlling kidney concentrating function and water conservation regulation as key factors in urine formation and extracellular volume homeostasis in humans with high salt intake.

Water conservation in the body through modification of urea osmolyte synthesis mechanisms in response to increased salt intake involves not only the kidney urea recirculation process but also activation of urea osmolyte synthesis in the liver and muscle tissue, as shown in a study in mice subjected to a high-salt diet (6). The study demonstrated that the urea content in the kidneys, liver, and muscles explains 87% of the variability in plasma urea levels in mice, with the liver contributing the most and the muscles the least. This indicates that the transport of urea osmolyte by the kidneys and the production of urea osmolyte in the liver and skeletal muscles are integrated physiological components. Considering the energy-intensive nature of urea synthesis, it has significant implications for energy metabolism with high salt intake, which can be observed under conditions of intensive diuretic therapy. Ishikawa et al. (22) showed that prolonged use of loop diuretics is associated with muscle wasting in patients with renal insufficiency.

The strengths of the present study are that it is the first to analyze the presence of an aestivation-like response in patients with chronic heart failure (CHF), depending on different levels of salt intake and the use of diuretic therapy. This expands upon prior findings by providing clinical evidence outside of the ICU setting. In the work of S. Nihlen et al. (5), the hypothesis of a similarity was presented between the human body’s response to fluid loss and the aestivation response in animals. The study included 241 post-intensive care patients and showed that the increase in total osmolality during the period of reducing body hyperhydration is due to loss of free water and changes in osmolyte balance: a decrease in the sodium contribution and an increase in the urea contribution. These results suggest an aestivation mechanism similar to the body’s response to prolonged dehydration, influencing patient survival. In an intensive care unit study from the third to the seventh day, 177 patients (73%) received furosemide. The analysis showed that the correlation between the proportion of urea and effective osmolality on the seventh day after the adjusted cumulative dose of furosemide was *r* = 0.55. Among patients with and without renal replacement therapy, this figure was *r* = 0.54 and *r* = 0.55, respectively. In the subgroup without furosemide and renal replacement therapy (*n* = 17), the correlation reached *r* = 0.92. For patients treated with furosemide but without renal replacement therapy, the figure was *r* = 0.61. There was a shift in the osmolytes from sodium (Na+) and potassium (K+) to urea, i.e., from inorganic to organic osmolytes. This phenomenon was also present in patients with normal estimated glomerular filtration rate (eGFR), even after adjusting the model for eGFR. Finally, although the level and dynamics of osmolality during the 3–7 days of ICU stay were similar between survivors and nonsurvivors, the urea-to-effective osmolality ratio was higher in patients who did not survive 90 days after ICU admission.

In 2022, Hultström et al. (23) conducted a study analyzing the effects of dehydration in patients with COVID-19, particularly examining the response to aestivation in the body and its impact on long-term disease outcomes. The results showed that metabolic aestivation in response to dehydration in patients with COVID-19 is associated with the severity of the disease. Metabolomic analysis identified amino acids with an aestivation profile, indicating muscle protein breakdown and the use of released amino acids for urea synthesis in the liver (24).

A study by C. Baumgartner et al. (25) examined the relationship between glycerophosphocholine (GPC), an organic osmolyte, and surrogate parameters of hydration status and osmolality in healthy individuals using non-invasive ^31^P-magnetic resonance spectroscopy of the calf and thigh muscles. In a sample of 30 volunteers, significant correlations were found between GPC levels and markers of fluid and electrolyte balance, such as uric acid (*r* = 0.437, *p* = 0.018) and urea (*r* = 0.387, *p* = 0.035). Multiple regression analysis revealed that GPC concentrations could predict changes in uric acid levels (R^2^ = 0.462, adjusted R^2^ = 0.421, *p* < 0.001), suggesting that the GPC content in skeletal muscles adapts in response to changes in fluid status.

The results of the present study demonstrated that changes in the contribution of individual osmolytes to plasma osmolality—particularly the increase in the proportion of urea—reflect the activation of water-conserving mechanisms, which play a key role in urine formation and the maintenance of extracellular fluid homeostasis in patients with chronic heart failure (CHF), especially under conditions of high salt intake and intensive diuretic therapy. Furthermore, the findings suggest that the altered contribution of urea to osmolality is not related to changes in renal function or more advanced CHF, but rather is a direct consequence of the effects of diuretic therapy on muscle tissue, leading to increased urea synthesis and a reduction in the proportion of sodium.

A study by G. Rossitto et al. (26) demonstrated that in patients with hypertension, a high sodium intake (>5 g/day) is associated with an increased glomerular filtration rate (127.5 mL/min/1.73 m^2^ vs. 94.1 mL/min/1.73 m^2^ with low sodium intake, *p* = 0.001) and increased renal energy expenditure for sodium reabsorption (difference of 18 kcal/day, *p* < 0.001), despite high fractional sodium excretion (0.81% vs. 0.39%, *p* < 0.001) and low water excretion (0.89% vs. 1.13%, *p* = 0.015). This leads to a catabolic shift and an increase in protein metabolism byproducts, which may elevate cardiovascular risk independently of blood pressure.

We hypothesized that the degree of aestivation might differ in patients on a diet with varying salt content undergoing intensive diuretic therapy. This is because patients on a long-term high-salt diet may have osmotically inactive sodium in muscle tissue (27). In our previous work (28), we found a relationship between Na + and negatively charged glycosaminoglycan (GAG) structures in rats on a high-salt diet. Therefore, we decided to investigate how excessive salt intake and intensive diuretic therapy in patients with CHF without ICU would affect the development of aestivation. In intensive diuretic therapy with sodium loss, partial replenishment can occur due to osmotically neutral sodium, so in our study, under a high-salt diet, the transition from inorganic (high sodium proportion) to organic osmolytes (high urea proportion) is more pronounced with a high-salt diet, whereas the effect is statistically insignificant with a diet containing less than 6 grams of salt per day. In our study, after 7 days of diuretic therapy, a statistically significant increase in the proportion of urea was observed (from 2.2% [1.8; 2.6] to 2.5% [1.9; 3.4], *p* = 0.15) and a decrease in the sodium proportion in plasma (from 46.4% [46.1; 46.8] to 46.3% [45.9; 46.7], *p* = 0.14) were observed. At the initial observation stage (first day), no statistically significant differences in plasma osmolality were found between the diuretic-receiving and nondiuretic-recipient groups.

To our knowledge, this is the first study to show that in patients who consume more than 10 grams of salt per day and undergo intensive diuretic therapy, urea increases significantly (*p* < 0.05) compared to other groups. However, with diets consuming 6 to 10 grams of salt per day and less than 6 grams, changes in the proportion of urea and sodium were statistically non-significant (*p* > 0.05). It is important to note that this is relevant in the context of hemodynamic changes, which are frequently observed and worsen the prognosis of patients with chronic heart failure (CHF). For instance, in the study by Sonaglioni A. et al. (29), heart failure patients exhibiting the “cold-dry” phenotype had significantly worse survival outcomes compared to other hemodynamic subtypes (*p* < 0.001), partly due to high sodium levels (HR 1.03, 95% CI 1.01–1.04). Consequently, the administration of diuretics to patients with the “cold-dry” hemodynamic profile should be approached with particular caution, and treatment should primarily aim to improve cardiac output and tissue perfusion.

## 5 Conclusion

The present study demonstrated that in patients with chronic heart failure (CHF), intensive loop diuretic therapy—particularly against the background of excessive sodium intake—is associated with changes in the plasma osmotic profile characteristic of metabolic aestivation. The key observation was a statistically significant increase in the contribution of urea and a decrease in the proportion of sodium to calculated plasma osmolality, indicating activation of water conservation mechanisms. These changes were not attributable to the severity of CHF or impaired renal function, as shown by the post–propensity score matching (PSM) analysis, and likely reflect osmolyte redistribution resulting from diuretic therapy and enhanced proteolysis.

From a clinical perspective, these findings suggest that monitoring the urea-to-osmolality ratio during the initial days of diuretic therapy may serve as an early biomarker for metabolic stress and muscle catabolism risk. Clinicians managing CHF patients should consider implementing individualized diuretic strategies that account for baseline dietary sodium intake, with particular attention to patients consuming >10 g/day of salt who may be at increased risk for aestivation-like metabolic responses. Early recognition of elevated urea-to-osmolality ratios should prompt consideration of nutritional support, physical therapy interventions, and potentially modified diuretic dosing regimens to achieve optimal fluid balance while minimizing catabolic consequences.

Thus, the findings offer a novel perspective on therapeutic strategies in the management of CHF. In particular, they highlight the importance of an individualized approach to prescribing diuretics that accounts for dietary sodium intake: high sodium consumption may exacerbate the adverse metabolic consequences of therapy by promoting a shift from inorganic to organic osmolytes and triggering skeletal muscle catabolism. This, in turn, may contribute to the development of sarcopenia and reduced functional capacity. The concept of metabolic aestivation may provide a new pathophysiological explanation for fatigue and weakness syndromes in such patients and warrants further investigation in clinical settings.

## 6 Limitations of the study

The present study has several limitations. It was conducted within a single medical center, which limits the generalizability of the findings to the broader population of patients with chronic heart failure (CHF). Despite the use of the propensity score matching (PSM) method, the final sample size was relatively small, reducing the statistical power of the analysis and precluding subgroup stratification. We did not perform direct measurements of muscle catabolism; thus, conclusions regarding increased proteolysis are based on indirect indicators (i.e., an increase in the proportion of urea in plasma osmolality). The study lacks long-term follow-up, so clinical outcomes such as changes in muscle mass, physical activity, and survival were not evaluated. The influence of other medications prescribed for heart failure—many of which may also induce diuresis and electrolyte disturbances—should also be investigated. Finally, the study did not assess direct markers of the hormonal stress response (e.g., cortisol, vasopressin, or ACTH), which could further clarify the underlying pathophysiological mechanisms.

Additionally, this study did not evaluate the clinical utility of the urea-to-osmolality ratio as a monitoring tool in routine clinical practice, nor did it assess the potential impact of implementing aestivation-aware diuretic strategies on patient outcomes such as length of stay, readmission rates, or functional status at discharge. Future prospective studies should investigate whether routine monitoring of osmolyte ratios and implementation of targeted interventions based on these findings can improve clinical outcomes and reduce the incidence of diuretic-associated muscle wasting in CHF patients.

## Data Availability

The raw data supporting the conclusions of this article will be made available by the authors, without undue reservation.
